# Economic Evaluation of Community Tuberculosis Active Case-Finding Approaches in Cambodia: A Quasi-Experimental Study

**DOI:** 10.3390/ijerph182312690

**Published:** 2021-12-02

**Authors:** Alvin Kuo Jing Teo, Kiesha Prem, Yi Wang, Tripti Pande, Marina Smelyanskaya, Lisanne Gerstel, Monyrath Chry, Sovannary Tuot, Siyan Yi

**Affiliations:** 1 Saw Swee Hock School of Public Health, National University of Singapore, National University Health System, Singapore 117549, Singapore; alvin.teo@aol.com (A.K.J.T.); kiesha.prem@nus.edu.sg (K.P.); ephwyi@nus.edu.sg (Y.W.); 2 Department of Infectious Disease Epidemiology, Faculty of Epidemiology and Population Health, London School of Hygiene and Tropical Medicine, London WC1E 7HT, UK; 3 McGill International TB Centre, Montreal, QC H4A 3S5, Canada; tripti.pande@hotmail.com; 4 Stop TB Partnership, 1218 Geneva, Switzerland; m.smelyanskaya@gmail.com; 5 KIT Royal Tropical Institute, 1092 AD Amsterdam, The Netherlands; L.Gerstel@kit.nl; 6 Cambodia Anti-Tuberculosis Association, Phnom Penh 12303, Cambodia; rath@thecata.org.kh; 7 KHANA Centre for Population Health Research, Phnom Penh 12301, Cambodia; tsovannary@khana.org.kh; 8 Faculty of Social Sciences and Humanity, Royal University of Phnom Penh, Phnom Penh 12150, Cambodia; 9 Department of Community and Global Health, Graduate School of Medicine, The University of Tokyo, Tokyo 113-0033, Japan; 10 Center for Global Health Research, Touro University California, Vallejo, CA 94592, USA

**Keywords:** tuberculosis, active case finding, passive case finding, Cambodia, cost-effectiveness, disability-adjusted life years

## Abstract

This study aimed to estimate the costs and incremental cost-effectiveness of two community-based tuberculosis (TB) active case-finding (ACF) strategies in Cambodia. We also assessed the number needed to screen and test to find one TB case. Program and national TB notification data from a quasi-experimental study of a cohort of people with TB in 12 intervention operational districts (ODs) and 12 control ODs between November 2018 and December 2019 were analyzed. Two ACF interventions (ACF seed-and-recruit (ACF SAR) model and one-off roving (one-off) ACF) were implemented concurrently. The matched control sites included PCF only. We estimated costs using the program and published data in Cambodia. The primary outcome was disability-adjusted life years (DALY) averted over 14 months. We considered the gross domestic product per capita of Cambodia in 2018 as the cost-effectiveness threshold. ACF SAR needed to test 7.7 people with presumptive TB to identify one all-forms TB, while one-off ACF needed to test 22.4. The costs to diagnose one all-forms TB were USD 458 (ACF SAR) and USD 191 (one-off ACF). The incremental cost per DALY averted was USD 257 for ACF SAR and USD 204 for one-off ACF. Community-based ACF interventions that targeted key populations for TB in Cambodia were highly cost-effective.

## 1. Introduction

Cambodia was one of the world’s 30 high tuberculosis (TB)-burden countries, with an estimated incidence of active TB of 274 (95% confidence interval [CI]: 177–392) per 100,000 population in 2020 [1]. The incidence of TB has gradually declined over the past two decades, along with improvement in TB treatment success rates and a reduction in TB-related mortality [2,3]. To fight TB, Cambodia has established an infrastructure network of TB service provision consisting of 100 district and referral hospitals and over 1140 health centers embedded in the national healthcare system [4]. The national TB program (NTP), led by the National Center for Tuberculosis and Leprosy Control (CENAT), also works closely with TB-affected communities, civil societies, non-governmental organizations such as KHANA and the Cambodia Anti-Tuberculosis Association (CATA), and other multilateral agencies to implement a comprehensive TB program. The program comprises all elements critical for TB control and elimination. The robust setup and the commitment by the Cambodian government to end TB has led to great successes in reducing the burden of TB disease and saw Cambodia transition out of the high TB burden list in 2021 [5]. Despite achieving great successes in TB control over the years, approximately 40% of the Cambodian population living with TB are undetected [2]. People with TB remain unreached and undiagnosed due to multifarious individual, social, community, and structural factors such as the lack of access to healthcare, residing in rural areas, lower education level, poor knowledge of TB, and stigma [6]. One way to effectively reach TB-affected communities is to actively seek people with TB and promptly link them to treatment and care through active case-finding (ACF) interventions [7].

To complement NTP’s efforts to end TB in Cambodia, KHANA, CATA, and CENAT have jointly implemented two community-based ACF approaches through a TB REACH-funded scale-up project (2018–2020). Both approaches aim to find undetected TB cases and promptly link them to care and treatment. The models have been previously evaluated with empirical evidence supporting their impact on increasing TB case detection. ACF using a seed-and-recruit (hereinafter ACF SAR) model was found to be associated with early initiation of TB treatment and increased detection of bacteriologically confirmed TB [8]. It was well-accepted by the affected communities and other key stakeholders in Cambodia [9]. One-off roving ACF (hereinafter one-off ACF) was effective in identifying older people with TB [10].

While there is mounting evidence demonstrating ACF’s effects on TB detection, there are limited economic evaluations conducted on the two models of interest [9,10]. Nonetheless, cost-effectiveness analyses of other ACF models in Cambodia have been previously conducted. A study in 2017 estimated that it would cost USD 249 to detect a TB case using a door-to-door strategy in poor urban areas of Phnom Penh, USD 308 to detect a TB case based on an ACF model among household and close contacts of people with TB, and USD 316 to detect a TB case using mobile screening units [11]. However, the relationship between expenditures and improvement in health was not evaluated in that study. A separate study evaluating an ACF model targeting households and close contacts suggested that the model was highly cost-effective with a cost per disability-adjusted life years (DALY) averted well below the gross domestic product per capita of Cambodia in 2014 [12]. While there has been empirical evidence on the cost-effectiveness of TB ACF strategies in Cambodia [11,12,13,14], community-based ACF interventions implemented in recent years, i.e., ACF using a seed-and-recruit model and one-off roving ACF targeting people aged ≥55 years, have yet to be rigorously examined. In this study, we conducted an economic evaluation of two community-based TB ACF strategies in Cambodia by assessing the costs and incremental costs per DALY averted among people with TB and considering the program and health system perspectives. We also evaluated the number of people with presumptive TB needed to be screened and tested to identify one TB case.

## 2. Materials and Methods

### 2.1. Study Setting and Population

**Figure 1:**
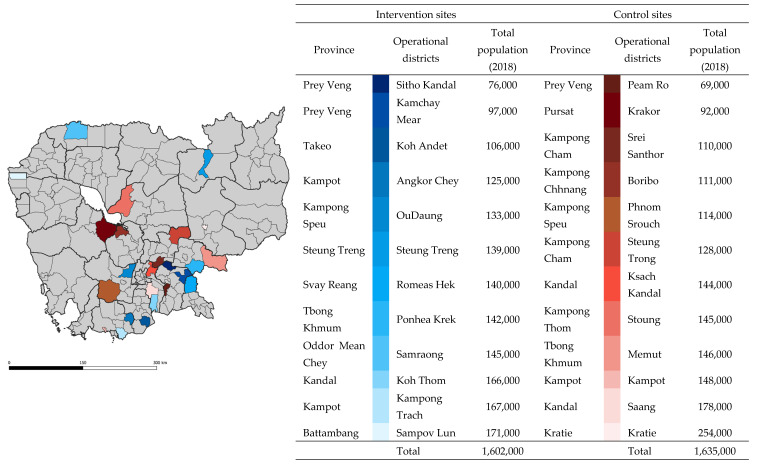
Location of study sites. Districts highlighted in shades of blue and red were intervention and control sites, respectively. Spatial data were extracted from Open Development Cambodia (2014). Light grey lines represent district borders, and black lines represent province borders. Total population data (2018) were rounded up to the nearest thousands.

We conducted an economic evaluation alongside a TB REACH program—a quasi-experimental study in 24 operational districts (ODs) across 15 provinces of Cambodia. The study compared the health impact and cost-effectiveness of two ACF modalities, implemented in 12 purposively selected intervention ODs, and a default passive case finding (PCF) in 12 control ODs matched by population size, urbanization, and TB burden. The characteristics of the sites are illustrated in Figure 1. People with presumptive TB and people with TB engaged by the different modalities in the screening, testing, referral, and diagnostic processes were included in the study.

**Figure 2:**
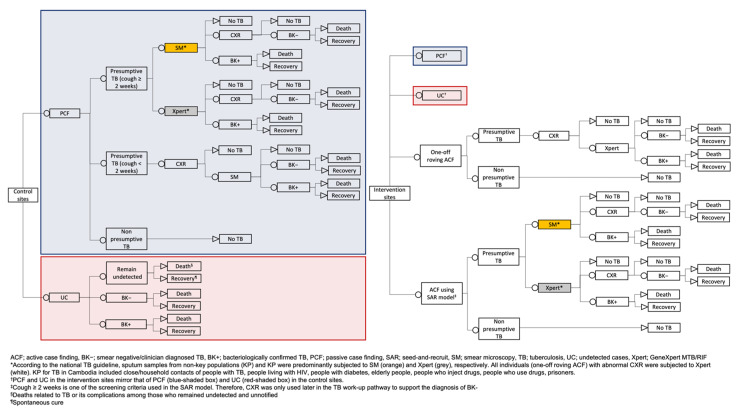
Decision tree for comparing the cost-effectiveness of ACF interventions (ACF using a seed-and-recruit model and one-off roving ACF) with control in 24 operational districts in Cambodia.

We obtained program and case notification data for ACF and PCF from the national TB surveillance system and program databases for analyses. Separately, we also used case notification data from 12 control ODs within the same period. All data were collected by the field staff or reported by the provincial health departments to the NTP. A decision tree (Figure 2) was used to guide the estimation of costs and the study’s outcome. The National Ethics Committee for Health Research Cambodia approved this study (112/NECHR).

### 2.2. Description of the ACF Models

The TB REACH program comprised two ACF models targeting key populations [8,15] implemented concurrently in the intervention sites. The models (Supplementary Figures S1 and S2) included (1) the ACF SAR model and (2) one-off ACF targeting people aged ≥55 years. The ACF SAR model was implemented by KHANA and one-off ACF by CATA concurrently in 12 intervention ODs between November 2018 and December 2019. TB diagnoses, treatment, and management were conducted following the national TB guidelines, irrespective of the case-finding models [16].

### 2.3. ACF SAR Model

The operations team from KHANA actively sought seeds, i.e., TB survivors and their TB-affected family members and other key informants in the community. The seeds were trained to find people with presumptive TB in the community, especially among key populations (KP) for TB (people aged 55 and above, people with diabetes, people living with HIV, household contacts of TB patients, and people who use and inject drugs) [17], using a symptom assessment questionnaire and refer those who might have TB to the health centers for TB workup. People newly diagnosed with TB were recruited to find other people with presumptive TB in the community in a snowball approach (Supplementary Materials). Sputum samples from people with presumptive TB were evaluated at the health centers using smear microscopy (non-KP) or GeneXpert^®^ MTB/RIF system (KP).

### 2.4. One-Off ACF

The model was implemented by CATA at a designated time, day, and place in the community (health centers or pagodas), targeting persons aged ≥ 55. To ensure sufficient coverage and reach, the one-off screening events were planned following the number of health centers and the proximity between villages in each OD and made known to the communities a priori. Symptom screening was conducted, and persons who exhibited TB symptoms and all individuals aged ≥ 55 regardless of symptoms were subjected to chest X-ray (CXR) examinations. Individuals with CXR suggestive of TB were further assessed using GeneXpert^®^ MTB/RIF system available on-site. People newly diagnosed with TB were referred for treatment and follow-up at the health centers.

### 2.5. Description of the PCF Model

PCF relies on a person with active TB in recognizing the symptoms, self-initiated care at a health facility, and the aptitude of health workers to initiate TB workup processes. Sputum samples from people with presumptive TB (member of KP) were collected for GeneXpert^®^ MTB/RIF examination. Sputum samples of non-KP were examined using smear microscopy. CXRs were performed for those who presented to the health centers with cough less than two weeks. Individuals with radiologic abnormalities were subjected to smear examinations. For those who were symptomatic but had a negative smear/GeneXpert^®^ MTB/RIF test, clinicians could make a smear-negative TB diagnosis based upon CXR and clinical findings [16].

### 2.6. Program and Health System Costs

#### 2.6.1. Costs of ACF Interventions

We estimated program and health system costs using a bottom-up approach. Program costs for both ACF models comprised categories associated with (1) human resources; (2) case-finding activities (operations and field workforce, project-related travels, logistics and setup, facilitation of referrals, meetings, and workshops, and information, education, and communication materials); (3) diagnostics and medical procedures (chest radiographs and sputum samples transportation) that incurred during the intervention period. We obtained program cost information from the implementers’ database.

As health system costs were not included in the program costing, we used primary cost data on personnel and diagnostics collected in 2014 (Table 1 and Supplementary Table S1) to estimate costs incurred at the health facilities [18]. The costs per test or procedure were computed based on the number of people with presumptive TB referred to the health facilities and the number of tests conducted. We also included the cost of treatment/community directly observed treatment, short course (C-DOTS) reported in 2014 [18]. TB treatment was initiated and managed by the public health facilities regardless of the mode of case-finding strategies. Therefore, the same treatment/C-DOTS cost applied to both ACF and PCF. The estimated costs incurred at the health facilities were adjusted for inflation using the consumer price index for Cambodia of 2018 [19] and added to the program cost to tally the total cost for the intervention.

Both program and health system costs were included in the cost-effectiveness analyses. A significant amount of health system costs was considered in evaluating the ACF SAR model as TB diagnoses were made at the public health facilities. For one-off ACF, health system costs were largely negligible because TB diagnoses were made on-site during the screening events.

#### 2.6.2. Cost of PCF

Due to the scarcity of primary cost data on PCF carried out during the study period, we estimated the costs of PCF by assuming that a similar proportion of people self-initiated care-seeking at the health facilities would undergo similar processes as per the people with presumptive TB referred by ACF SAR model to the health facilities for TB workup.

Of the 12074 people with presumptive TB identified by the ACF SAR model, all individuals received clinical consultation and examination at the public health facilities. An equal proportion of the individuals received either GeneXpert MTB/RIF or smear microscopy to diagnose TB. After factoring in the number of liquid cultures and follow-up work on positive culture results, drug susceptibility testing for multi-drug resistance TB, the total additional cost incurred at the health facilities for diagnostic and medical procedures (health system) was USD 234,931 (Supplementary Table S1). The costs of additional chest radiographs and sputum samples transportation (total: USD 12,955) were budgeted in the program costs, but these processes were part of the post-referral pathway that took place at the health centers. Therefore, the total expenditures to diagnose a TB case referred by the ACF SAR model at the public health facilities were estimated to be USD 247,886.

We computed the average cost to perform TB workup per person at the health facilities, including the costs for chest radiographs and transportation of sputum samples (USD 20.5, Supplementary Materials). Subsequently, we multiplied the cost by the number of TB cases notified by PCF in the intervention and control sites to estimate PCF costs. Treatment/C-DOTS costs were also included. We multiplied the estimated treatment cost per person (USD 65.4) [18] with the total number of TB cases notified by PCF. Due to the lack of primary data, we assumed that those notified via PCF initiated and remained in TB treatment for 6 months. In this study, we did not include other fixed costs not included in the computation of health system and program costs, indirect medical costs, and other non-medical costs.

For both ACF and PCF, we considered costs incurred from the planning and implementation of case-finding activities to the point where TB treatment was completed. However, we lacked data on treatment adherence and completion in this study. Therefore, in the Cambodian context where treatment success was relatively high (94%) [20], we assumed that the costs of 6-months TB treatment applied to all who initiated it. All costs were presented in United States dollars (USD) in 2018.

### 2.7. Study Outcomes

The primary outcome was the incremental cost-effectiveness ratio (ICER) [21]—incremental cost per DALY averted among people with TB in the intervention sites compared to the control sites. We defined DALY based on the World Health Organization (WHO)’s metrics of quantifying the burden of disease—DALY as the sum of the years of life lost (YLL) due to premature death and the years lost due to disability (YLD) for people living with the disease [22].

The secondary outcomes were the cost per all-form TB cases identified, cost per bacteriologically confirmed TB cases, and the number of people with presumptive TB that needed to be screened (NNS) and tested (NNT) to find one TB case—both all-form and bacteriologically confirmed TB cases—in the intervention sites.

### 2.8. Cost-Effectiveness Model Descriptions and Assumptions

We calculated DALYs for TB cases identified by the two ACF models in the intervention sites and cases identified by PCF in both the intervention and control sites. While the ACF models were implemented concurrently, we assumed that they were independent of each other. To estimate ICERs, we computed the DALYs for the remaining TB cases undetected in the control sites during the study period to project the DALYs that could have been averted, attributable to the respective ACF interventions.

### 2.9. Years Lost due to Disability (YLD)

We used the total number of TB cases reported from the intervention sites (ACF SAR model, one-off ACF, and PCF) and the estimated total number of cases in the control sites (cases notified by PCF and undetected TB cases) to calculate the respective YLD. TB morbidities were estimated using TB specific disability weights of 0.33 (95% CI 0.22–0.45) and 0.41 (95% CI 0.27–0.55) for HIV-negative and HIV-positive populations [23].

We estimated the number of undetected TB cases in the control sites by assuming that the cases identified by ACF and PCF would make up the total TB prevalence within reasonable bounds in the selected sites. As the number of cases notified by PCF was comparable between both sites, the undetected TB cases in the control sites would reflect the cases detected by ACF models in the intervention sites. We tested this assumption by comparing the total TB cases notified to CENAT from the intervention sites (ACF and PCF) with the model-forecasted TB prevalence [24] in the same sites in 2019. The total cases notified (ACF and PCF) from the intervention sites were well within the lower and upper plausible bounds of the number of TB cases projected in 2019 under three different scenarios (no future improvement, continual reduction, and gross domestic product (GDP) projection) [24].

The calculations involved four major steps as follows: (1) We regarded the case notification data obtained from CENAT for the 12 intervention ODs represented the total number of TB cases identified by PCF and the two ACF interventions. (2) We calculated the proportions of cases notified by PCF and ACF interventions. (3) Subsequently, we used the median proportion of cases presumably notified by PCF in the intervention sites (0.384, Supplementary Materials) to estimate the total number of TB cases in the control sites. (4) Finally, the number of undetected TB cases that could have been averted by the interventions in each control OD was determined by the difference between the total number of TB cases and cases notified by PCF.

Nonetheless, we could not account for the remaining undetected cases in the intervention sites despite ACF implementation. As the number of undetected cases in the control sites was estimated based on data from the intervention sites, we regarded that a similar number of cases would remain undetected in both groups. Thus, the remaining undetected cases would cancel out during the computation of DALYs.

### 2.10. Years of Life Lost (YLL)

We computed YLL due to TB deaths in the intervention sites using the WHO life tables for Cambodia [25]. We extended the proportion of TB deaths reported by PCF in the control sites (32 deaths for 2875 TB cases (1.11%)) to approximate the TB deaths that would have occurred among the undetected TB cases in the same localities. As such, we did not account for deaths that occurred among those who remained undetected and unnotified. Due to the unavailability of information on the individual’s age and sex who experienced TB death in the control sites, we used a previously reported average age of TB death [26] and standard life expectancy [27] in Cambodia to calculate YLL.

### 2.11. Data Analyses

#### 2.11.1. Primary Analyses

We evaluated the comparability between the intervention and control sites based on 3 parameters (historical TB notification data, TB cases notified passively during the study period, and the number of TB cases notified in the first quarter post-intervention) using the Student’s *t*-test and Mann–Whitney test. We calculated ICER comparing the intervention and control groups: (1) ACF SAR model + PCF (intervention sites) vs. PCF (control sites) and (2) one-off ACF + PCF (intervention sites) v. PCF (control sites). The key parameters for cost-effectiveness analyses are presented in Table 1.

We considered an intervention to be cost-effective if the ICER was lower than the GDP per capita [28,29] of Cambodia—USD 1643 [30]. We compared the cost-effectiveness ratios to a tighter Cambodia-specific threshold (USD 297; adjusted to 2018 USD [31]) developed by Ochalek and colleagues [32]. We also computed the cost per TB case diagnosed (all-forms and bacteriologically confirmed TB) for each ACF intervention. We calculated the number of people with presumptive TB needed to be screened and tested to find one TB case for each ACF intervention by dividing the number of persons screened and tested by the number of TB cases detected.

#### 2.11.2. Sensitivity Analyses

We conducted one-way and probabilistic sensitivity analyses to account for the uncertainty around our assumptions and published cost estimates. For the costs associated with PCF, we estimated the uncertainties of the PCF costs used in the primary analyses and investigated two variants using the costs of PCF published separately in Cambodia in 2012 [33] and 2014 [12] (Supplementary Materials). To estimate the 95% uncertainty interval (UI) for PCF cost estimates and costs of treatment/C-DOTS, we generated 1000 draws from a log-normal distribution using the costs per person as the means, and we assumed that the standard deviation (SD) was 20% of the means. The assumption, however, was arbitrary as we lacked data to compute the SD of our estimates. Nevertheless, we expected that the resultant 95% confidence interval (CI) would cover the reasonable range of cost per DALY averted. The 2.5th and 97.5th percentile of the simulated data formed the lower and upper bound, respectively. For costs associated with the estimation of diagnostic and medical procedures (health system), we presented the ICER using the published minimum and maximum values.

For the calculation of YLL and DALY in the control sites, we explored other scenarios where we varied the number of additional TB deaths that could have occurred among the undetected TB cases in the control sites. We assumed zero TB deaths among the undetected cases in the control sites as the minimum value. Using the estimated number of undetected TB deaths in the control sites (n = 51; as presented in the primary analyses), we assumed inflation of 2 times as the maximum number of deaths that could have occurred among the undetected cases. We generated 1000 draws from a standard uniform distribution generated using the respective minimum and maximum values to estimate the uncertainty interval (UI) with the 2.5th percentile of the distribution as the lower bound and the 97.5th percentile as the upper bound.

For the estimation of undetected cases in the control sites in the one-way sensitivity analysis, we assessed the reliability of our analyses by bootstrapping the proportions of cases detected via PCF (Supplementary Table S2) in the intervention sites with 1000 replications. The corresponding number of undetected TB cases in the control sites that could have been averted by the interventions is presented in Supplementary Table S3. The bootstrapped median and percentile UI (2.5th and 97.5th) were reported.

We also examined the change of ICERs using the lower and upper bound of the disability weights included in DALY’s calculations. The ICERs, if interventions were not implemented in tandem, were also explored (Supplementary Materials).

All analyses were conducted using Microsoft Excel 365 ProPlus (Microsoft, Redmond, WA, USA), STATA 14 (Stata Corp LP, College Station, TX, USA), and R (R Foundation for Statistical Computing, Vienna).

## 3. Results

### 3.1. Comparison of Intervention and Control Sites

There were 1,602,000 people residing in the intervention sites and 1,635,000 in the control sites. There was also an equal number of urban and rural Ods in the two groups. We also investigated historical TB case notification data from 2016 to 2018 and did not find a statistical difference between intervention and control sites (*p =* 0.769). The number of historical TB case notification by year also did not differ significantly (2016: *p* = 0.908; 2017: *p* = 1.000; 2018: *p* = 0.356) between the intervention and control sites. The number of TB cases notified passively in the two groups during the study period was comparable (*p* = 0.070). Similarly, no statistically significant difference was observed in the number of TB cases notified in the first quarter post-intervention (January 2020 to March 2020) between intervention and control sites (*p* = 0.435).

### 3.2. TB Case Detection, the Number People with Presumptive TB Needed to Be Screened and Tested to Find One Person with TB

Between November 2018 and December 2019, the seed-and-recruit model screened 21,539 individuals and referred 12,074 individuals for TB workup. The model identified 1577 all-form TB cases, of which 438 were bacteriologically confirmed. To find one bacteriologically confirmed TB case, the model needed to screen 48.6 and test 27.4 individuals with presumptive TB. In total, the one-off roving ACF screened 189,865 individuals and referred 51,636 for TB workup. The one-off roving ACF identified 2303 all-form TB cases, of which 648 were bacteriologically confirmed. The model needed to screen 289 and test 78.6 individuals with presumptive TB to find one bacteriologically confirmed TB case (Table 2).

In the same period, 2191 (intervention) and 2875 (control) TB cases were notified by PCF. We estimated that there were 4615 undetected TB cases in the control sites. Among people with TB recruited via the ACF SAR model and one-off ACF, 7 and 13 TB deaths were reported. In the control sites, 32 TB deaths were notified. We estimated that an additional 51 TB deaths would have occurred among the undetected cases.

### 3.3. Costs of Interventions and Control

Case-finding activities, human resources, and administrative, diagnostics, and medical procedures comprised the bulk of the interventions’ costs (Table 3). The total program cost to implement the ACF SAR model was USD 487,631. We also included other costs incurred at the health facilities to complete the TB diagnosis process and treatment, and this amounted to USD 824,504. By contrast, the program cost to carry out the one-off ACF was USD 440,755. As most TB cases were diagnosed in situ, the additional diagnostic costs at the health facilities were negligible. After accounting for treatment costs, the health system cost amounted to USD 587,949. Based on the number of TB cases notified to CENAT, we estimated the cost of PCF to be USD 188,158 in the intervention and USD 246,898 in control sites.

### 3.4. Cost per TB Diagnosis, DALYs, and ICER

The program and health system costs to diagnose one TB (all-forms) case and one bacteriologically confirmed TB case were USD 458 and USD 1631, respectively, for the ACF SAR model. For one-off ACF, the corresponding costs were USD 191 and USD 671, respectively. The DALYs for persons with TB identified by ACF SAR and one-off ACF in the intervention sites were 620.5 and 1001.7, respectively. In the control sites, we estimated that the undetected TB cases resulted in 2723.5 DALYs (Table 4). Compared to the control sites, the estimated ICER was USD 257 for the ACF SAR model and USD 204 for the one-off ACF (Table 4).

### 3.5. Sensitivity Analyses: Costs of PCF, Diagnostics and Medical Procedures, and Treatment/C-DOTS

**Figure 3:**
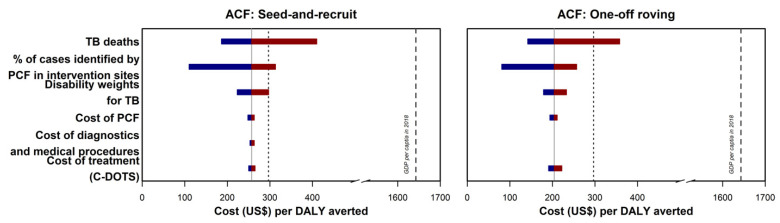
Incremental cost-effectiveness ratio (ICER) tornado plot for multiple one-way sensitivity analyses. TB deaths referred to the estimated TB-related mortality among the undetected cases in the control sites. The percentage of cases identified by PCF in the intervention sites were used to estimate the number of undetected TB cases in the control sites. Disability weights were used in the calculation of years lived with disability and disability-adjusted life years. Cost of PCF referred to the cost used in the primary analysis. Cost of diagnostics and medical procedures referred to the data used to estimate health system cost in the main analysis. The corresponding ICER was not presented for one-off ACF as TB diagnoses were made at the program sites and the costs of the diagnostic were negligible from the health system perspective. Cost of treatment (C-DOTS) referred to the costs estimated from a study by Yadav and colleagues. The grey lines represent the ICER presented in the primary analysis. The cost-effectiveness thresholds—gross domestic product per capita of Cambodia in 2018 and country-specific threshold estimated by Ochalek and colleagues—are presented using black dashed lines and dotted lines, respectively. The maroon section of the bars corresponded to values above the reference ICER, and the blue section of the bars correspond to values below the reference ICER.

After accounting for the uncertainties of the PCF cost used in the primary analysis, the ICERs for the ACF SAR model and one-off ACF were USD 257 (95% UI 248–263) and USD 204 (95% UI 194–217), respectively (Figure 3). The ICERs remained analogous when other variants of previously published PCF costs were analyzed (Supplementary Table S4). Similarly, the ICERs for the two models were below the cost-effectiveness thresholds upon considering the variations in diagnostics and medical procedures, and C-DOTS cost (Figure 3). We reported a similar finding when an assessment was made using another C-DOTS cost published previously (Supplementary Materials).

### 3.6. Sensitivity Analyses: Variation of YLL, Undetected Cases in the Control Sites, and Disability Weights

The ICERs for the ACF SAR model and one-off ACF were USD 257 (95% UI 186–410) and USD 204 (95% UI 140–358), respectively, after considering the additional TB deaths among the undetected cases in the range of zero to 102 (Figure 3). Likewise, the ICERs were below the cost-effectiveness threshold using the bootstrapped estimates in the computation of undetected cases in the control sites and the lower and upper bounds of the disability weights (Figure 3).

### 3.7. Probabilistic Sensitivity Analyses: Costs of PCF, Diagnostics and Medical Procedures, and C-DOTS, Variation of YLL, Undetected Cases in the Control Sites, and Disability Weights

Out of the 1000 probabilistic runs, almost all (>99%) simulated ICERs for the ACF SAR and one-off ACF models were below the 2018 GDP per capita (USD 1643); the results are presented in the Supplementary Materials. When judged against the more conservative country-specific cost-effectiveness threshold (USD 297), [32] ACF SAR and one-off ACF models were cost-effective with probabilities 0.804 and 0.862, respectively (Supplementary Materials).

## 4. Discussion

This economic evaluation study found that the community-based TB ACF approaches—ACF SAR model and one-off ACF—were cost-effective. The ICERs for both models are well below the GDP per capita in 2018 (USD 1643) and the country-specific cost-effectiveness thresholds (upper end: USD 297) that were estimated by Ochalek and colleagues [32]. In Cambodia, one ACF modality targeted household and symptomatic neighborhood contacts of people with TB has been reported to be highly cost-effective with a cost per DALY averted of USD 330 in 2013 [12]. A review that evaluated cost-effectiveness analyses of TB ACF conducted across different settings and geographical locations in 2016 concluded that community-wide ACF could be highly cost-effective in settings with a high incidence of TB [34]. Targeted screening and case-finding interventions among high-risk populations such as close contacts of TB patients, people living with HIV, and prisoners in such settings were also cost-effective [34]. Our findings further supported the notion that TB community-based ACF strategies are cost-effective, especially in high TB burden settings.

Our approach in estimating the cost of PCF in the comparator is predominantly conservative; we estimated the cost of PCF based on the actual number of TB cases notified instead of the number of people screened and tested. That would have led to the difference in cost between intervention and control to remain significant and marginally inflate the ICER. Our sensitivity analyses included the assessment of other potential scenarios to appraise the rigor of the findings. We considered more liberal estimates concerning the cost of PCF using data from previously published studies. However, as the number of TB cases notified by PCF was comparable in both intervention and control sites, the effect of the cost was mostly offset, leading to minor changes in the final ICER estimates. We also investigated another conservative circumstance in the control sites where no additional TB deaths were recorded among the undetected TB cases. With these variations in place, the ICER estimates and their Uis were lower than the pre-defined threshold, further supporting our findings that both ACF models were cost-effective. Nevertheless, further optimization of the intervention, especially the ACF SAR model (higher ICER of the two), is necessary by improving the technical capacities of field staff, seeds, and recruiters and continuous engagements with the community to improve its cost-effectiveness.

In this study, the cost per all-form TB case diagnosed was USD 458 (ACF SAR model) and USD 191 (one-off ACF). The latter was an approach that has been routinely implemented and previously evaluated in Cambodia, where the costs per TB case diagnosed were reported to be USD 316 [11] and USD 156 [13] in two separate studies. Another modality that has been evaluated in Cambodia was an approach that targeted household and symptomatic neighborhood contacts. It was estimated that it would cost USD 308 to diagnose one TB case [11]. A strategy that is similar in principle (household contact investigation) implemented as part of a randomized controlled trial in Vietnam reported the cost per TB case diagnosed was USD 181 [35]. The variation in costs could be attributed to the strategic and methodologic differences in cost optimization (such as the deployment of community volunteers) and operational framework. A direct comparison of the two ACF models evaluated in this study showed that it would cost less to find one TB case in the community via the one-off ACF model. The mass screening element of the approach could have resulted in economies of scale yielding more TB diagnoses at a lower cost.

Globally, a systematic review in 2013 reported that the NNS for strategies that targeted specific facilities or risk groups were lower than community/population-wide approaches [36]. In Cambodia, ACF models that focused primarily on TB risk groups have also been shown to promote shorter delays to health-seeking and improve TB case detection [8,14]. The two models evaluated in this study showed differing efficiency in detecting different numbers of TB cases among the populations screened and tested. Despite incurring a higher cost, the ACF SAR model that targeted TB KPs [15] in the communities recorded a lower NNS and NNT than the one-off ACF. Nevertheless, the NNS for both models were comparable and lower than the NNS reported in case-finding strategies targeting different risk groups in high TB incidence countries [36], suggesting optimal TB case detection performance. While our findings generally corresponded to the NNT reported by ACF models previously implemented in Cambodia, the NNT for the ACF SAR model, in particular, was lower in comparison to the two studies [37,38]. The empowerment that people with presumptive TB received from their social contacts in the community has been reported as an enabler to TB care-seeking in Cambodia [6]. Therefore, an ACF strategy that capitalized on social networks and community mobilization to target KPs, such as the ACF SAR model, might explain the lower level of testing efforts required to detect one TB case.

Despite the lack of randomization, the case notification data (historical and in the first quarter after the interventions have ceased) and cases notified by PCF during the intervention period were comparable between intervention and control sites. The latter also suggested minimal disruption in the trend of TB cases notified through PCF despite ACF interventions. Thus, the crowding-out effect (cases detected by PCF if ACF interventions were not implemented) in this study was nominal. One potential limitation of our study was that both interventions were implemented in the same sites. Therefore, we were unable to compare the two interventions against each other. Notwithstanding the possibility of concurrent operations that might dilute the true effect each ACF model could have on TB case detection, our sensitivity analyses showed that the ICER estimates were below the cost-effectiveness threshold (Supplementary Materials). We were also unable to quantify the combined effects of the two synchronous interventions. The pros and cons of such a strategy would have to be further investigated. Similarly, we have also yet to conduct a comparative analysis of benefits between the ACF SAR model and one-off ACF, and therefore, further research is warranted.

The disability weights currently available did not account for the effect of early and delayed diagnoses by considering that the time to diagnosis was similar across the board, potentially biasing the estimates towards approaches that did not effectuate earlier TB diagnosis. Fine-tuning of the disability weights to account for the effects of early and delayed diagnoses would help provide more robust estimates of the cost-effectiveness of ACF strategies. We also lacked data granularity on several parameters. First, there was a paucity of disaggregated data on PCF, which might have affected the accuracy of our estimates. Second, we did not have information on TB types, limiting this cost-effectiveness analysis to all-form TB in adult populations. Third, we were only able to utilize retrospective programs and health system costs. While we have strived to utilize the available information fully, we acknowledged the lack of detailed individual-level data on expenditures (direct and indirect medical costs and other non-medical costs) incurred in the pathway leading to TB diagnoses. Therefore, we have undertaken pragmatic approaches in the analyses to not overcompensate for poor quality cost data [39]. While we have accounted for treatment costs in our analyses, we were unable to account for other direct medical costs that may have been incurred during treatment, such as the management of adverse effects. Lastly, we did not account for age in our analyses. Operationally, one-off ACF targeted older persons, which could have led to higher mortality among people with TB [40] identified by the intervention resulting in a higher DALY. While it is plausible that if the intervention is extended to target younger people with TB with a lower risk of mortality, the intervention would remain cost-effective. Nevertheless, we lacked information on how one-off ACF would perform if younger individuals were targeted.

In this study, we found that if policymakers are willing to pay USD 297 per DALY averted, the probability of both ACF activities being cost-effective is >80% and a willingness-to-pay threshold of <USD 500 would render the probability of the ACF SAR and one-off ACF to approximately 90%. In the study by Yadav and colleagues, the probability of ACF activities being cost-effective is >90% if the policymakers are willing to pay USD 600 per DALY averted [12]. With increased domestic funding, commitment and the political will to end TB in Cambodia [20,41], the willingness-to-pay is likely to increase over time, further supporting that the two ACF models evaluated in this study would be cost-effective from the policymakers’ perspective. Nevertheless, continuous engagements with the NTP are warranted to further inform policy decision-making and case-finding activities.

To our knowledge, this is the first cost-effectiveness analysis of two community-based ACF models—ACF using a seed-and-recruit model and one-off roving ACF targeting people aged ≥55 years—in Cambodia, a high TB-burden country. This study provides practical information necessary for evaluating TB case-finding activities in Cambodia and making decisions on program expansion and scale-up. We found that the ACF SAR model and the one-off ACF targeting persons aged ≥ 55 were cost-effective. Thus, our findings further supported the need for targeted engagements with KPs (modus operandi of these ACF interventions) to find missing people with TB in the community. While the ICERs differed between the two models, other effectiveness measures such as shorter delays to diagnosis and treatment, identification of bacteriologically confirmed TB, and the number of people with presumptive TB needed to be screened and tested should be considered during expansion and scale-up [8]. Furthermore, the different dimensions of effectiveness should be systematically measured and considered within a more robust research framework, such as a randomized controlled trial that allows a direct comparison between different ACF modalities [15].

## Figures and Tables

**Table 1:** Key parameters in cost-effectiveness analysis.

Parameters	Best Estimate	Upper Bound	Lower Bound	Comments	References
Epidemiology and DALY determinants					
Incident cases identified by ACF SAR	1577			Primary data	
Incident cases identified by one-off ACF	2303			Primary data	
Incident cases identified by PCF (intervention sites)	2191			Primary data	
Incident cases identified by PCF (control sites)	2875			Primary data	
Other undetected cases in the control sites	4615			Difference between the total number of TB cases (estimated) and cases notified by PCF ^†^	
Number of people living with HIV	6			Primary data	
Disability weights (HIV positive)	0.41	0.27	0.55		[23]
Disability weights (HIV negative)	0.33	0.22	0.45		[23]
Number of TB deaths					
ACF SAR	7			Primary data	
One-off ACF	13			Primary data	
PCF (intervention sites)	4			Primary data	
PCF (control sites)	32			Primary data	
Among other undetected cases (estimated)	51			Estimated from the proportion of TB deaths reported by PCF in the control sites ^††^	
Premature years of life lost	Expectation of life at age *x*			*x* = age of people with TB who died (primary data). Premature years of life lost based on WHO life tables for Cambodia 2019	[25]
Mean age of people with TB who died	46.3				[26]
Standard life expectancy	69.57			Standard life expectancy at birth in 2018	[27]
Cost estimates					
Program cost (human resources, case-finding activities, diagnostics, and medical procedures)					
ACF SAR (USD) *	487,631			Primary data	
One-off ACF (USD)	440,756			Primary data	
Health system costs					
GeneXpert (USD)	39.0	40.8	37.3	Published data	[18]
Clinical exam (USD)	1.7	2.0	1.3	Published data	[18]
Chest X-rays (USD)	2.3	2.4	2.2	Published data	[18]
Fluorescent smear microscopy (USD)	1.9	2.1	1.7	Published data	[18]
Ziehl–Neelsen smear microscopy (USD)	1.4	1.4	NA	Published data	[18]
Liquid culture (USD)	19.0	25.1	12.8	Published data	[18]
Follow-up work on positive culture results and identification of MTB complex (USD)	14.4	16.2	12.6	Published data	[18]
Drug susceptibility testing for MDRTB (USD)	48.7	48.7	NA	Published data	[18]
Specimen transport (USD)	3.6	5.7	1.5	Published data	[18]
C-DOTS/TB treatment (USD)	65.0	74.0	56.7	Published data	[18]
C-DOTS/TB treatment (USD)	250	300	200	Published data. Included in sensitivity analysis.	[12]
PCF cost (USD)	75.5	111.0	50.4	Published data. Upper and lower bounds estimated from a log-normal distribution. Standard deviation 20% of the means assumed. Included in sensitivity analysis.	[12]
PCF cost (USD)	49.6	71.8	33.1		[33]

DALY: disability-adjusted life years, ACF SAR: active case finding using the seed-and-recruit model, one-off ACF: one-off roving active case finding, PCF: passive case finding, HIV: human immunodeficiency virus, WHO: World Health Organization, USD: United States dollar, TB: tuberculosis, C-DOTS: community directly observed treatment, short course, NA: not available. * USD reported were adjusted for inflation using the consumer price index for Cambodia of 2018 and presented in USD 2018. ^†^ Calculations involved four major steps: (1) We regarded the case notification data obtained from CENAT for the 12 intervention sites represented the total number of TB cases identified by PCF and the two ACF interventions. (2) Then, we calculated the proportions of cases notified by PCF and ACF interventions, respectively. (3) Subsequently, we used the median proportion of cases presumably notified by PCF in the intervention sites (0.384) to estimate the total number of TB cases in the control sites. (4) Finally, the number of undetected TB cases that could have been averted by the interventions in each control OD was determined by the difference between the total number of TB cases and cases notified by PCF. ^††^ Proportion of TB deaths reported by PCF in the control sites (32 deaths for 2875 TB cases (1.11%)) to approximate the TB deaths that would have occurred among the undetected TB cases in the same localities.

**Table 2:** TB active case-finding program outputs.

	ACF Using A Seed-and-Recruit Model	One-Off Roving ACF
The number of individuals screened for symptoms suggestive of TB or eligibility for referral and TB tests	21,539	189,865
The number of individuals referred and tested for TB	12,074	51,636
The number of TB (all-forms) cases detected	1577	2303
The number of bacteriologically confirmed TB cases detected	443	657
The number of people with TB (all-forms) who initiated treatment	1560	2251
The number of TB deaths reported	7	13
The number needed to screen to find 1 TB (all-forms) case	13.7	82.4
The number needed to screen to find 1 bacteriologically confirmed TB case	48.6	289.0
The number needed to test to find 1 TB (all-forms) case	7.7	22.4
The number needed to test to find 1 bacteriologically confirmed TB case	27.4	78.6

TB: tuberculosis, ACF: active case finding.

**Table 3:** Program and health system costs elements of TB active case-finding programs.

	ACF Using a Seed-and-Recruit Model	One-Off Roving ACF
ACF Program	USD	USD
Human resources	89,680	76,751
Case-finding activities—intervention implementation and field workforce, project-related travels, logistics and setup, facilitation of referrals, meetings and workshops, and information, education, and communication materials	347,267	211,516
Diagnostics and medical procedures	12,955 *	97,519
Administrative	37,729	54,970
Total (program)	487,631	440,755
Health system	USD	USD
GeneXpert MTB/RIF	205,488	0
Consultation and clinical examination	20,313	0
Smear microscopy (fluorescent or Ziehl–Neelsen)	8821	0
Liquid culture and other follow-up work on positive culture results and identification of Mycobacterium tuberculosis complex	67	0
Drug susceptibility testing for individuals suspected of drug-resistant TB	243	97
Treatment/C-DOTS	101,941	147,096
Total (health system)	336,873	147,193
Total (program and health system)	824,503	587,948

TB: tuberculosis, ACF: active case finding, USD: United States Dollar; MTB/RIF: Mycobacterium tuberculosis/resistance to rifampicin. * Costs for additional chest radiographs and sputum samples transportation.

**Table 4:** Incremental cost-effectiveness ratio of ACF models.

	Total Costs (USD)	Total TB Cases	Total TB Deaths	YLL	YLD	DALY	Cost (USD) per DALY Averted
Intervention sites							
ACF SAR	722,562	1577	7	92.6	525.4	620.5	257
One-off ACF	440,853	2303	13	238.5	766.9	1001.7	204
PCF	188,158	2191	4	93.1	729.6	822.7	
Control sites							
Undetected TB cases		4615	51	1186.8	1536.7	2723.5	Reference group
PCF	246,898	2875	32	744.6	957.4	1702.0	

TB: tuberculosis, YLL: years of life lost, YLD: years lost due to disability, DALY: disability-adjusted life years, PCF: passive case finding, ACF SAR: active case finding using a seed-and-recruit model, one-off ACF: one-off roving active case finding, USD: United States Dollar.

## Data Availability

The data used in this study are available on request to the corresponding author, subject to the approval of the National Ethics Committee for Health Research Cambodia.

## Supplementary Materials

The following are available online at https://www.mdpi.com/article/10.3390/ijerph182312690/s1, Figure S1: Description of the active case finding using a seed-and-recruit model, Figure S2: Description of the one-off roving active case-finding model, Table S1: Breakdown of health system cost: diagnostic and medical procedures, Table S2: Estimated proportion of TB cases detected via PCF in the intervention sites, Table S3: Estimated number of undetected cases in the control sites that could have been identified by ACF if implemented, Table S4: Sensitivity analyses of the different costs of PCF and the resultant incremental cost-effectiveness ratio, Figure S3: Distribution of TB cases identified during the implementation period, Figure S4: Probabilistic sensitivity analysis: Incremental cost-effectiveness scatter plots and acceptability curves of two active case-finding interventions, seed-and-recruit and one-off roving, against the default passive case-finding model.
