# Supplementary material for: Economic Evaluation of Community Tuberculosis Active Case-Finding Approaches in Cambodia: A Quasi-Experimental Study
Source: Int J Environ Res Public Health. 2021 Dec 2;18(23):12690. doi: 10.3390/ijerph182312690 (PMC8656683; doi:10.3390/ijerph182312690)
Supplement: Supplementary file 1 [file ijerph-18-12690-s001.zip › ijerph-1415257-supplementary.pdf]

Economic evaluation of community tuberculosis active case-finding approaches in Cambodia: a quasi-experimental study

Supplementary materials

Active case-finding models 2

Supplementary Figure S1: Active case finding using a seed-and-recruit model.... 2

Supplementary Figure S2: One-off roving ACF. 3

Cost per test or sample incurred at the health centers (diagnostic and medical procedures) 4

Supplementary Table S1: Breakdown of the health system cost: diagnostic and medical procedures. 4

Estimation of the number of undetected cases in the control sites 5

Supplementary Table S2: Estimated proportion of TB cases detected via PCF in the intervention sites. 5

Supplementary Table S3: Estimated number of undetected cases in the control sites that could have been identified by ACF if implemented. 5

Costs of PCF and C-DOTS 6

Cost of PCF used in the main analysis 6

Costs of PCF estimated from published studies 6

Supplementary Table S4: Sensitivity analyses of the different costs of PCF and the resultant incremental cost-effectiveness ratio. 7

Cost of C-DOTS 7

Sensitivity analysis of the variation of disability-adjusted life years of the ACF models if the models were not implemented concurrently and the resultant incremental cost-effectiveness ratio 8

Supplementary Figure S3: Distribution of TB cases identified during the implementation period. .. 8

Supplementary Figure S4: Probabilistic sensitivity analysis: Incremental cost-effectiveness scatter plots and acceptability curves of two active-case finding interventions, seed-and-recruit and one-off roving, against the default passive case-finding model. 9

References 10

Active case-finding models

Supplementary Figures S1 and S2 (adapted from Teo et al, 2020) [1] illustrates the operational procedure for implementing active case finding using a seed-and-recruit model and one-off roving ACF.

Supplementary Figure S1: Active case finding using a seed-and-recruit model.

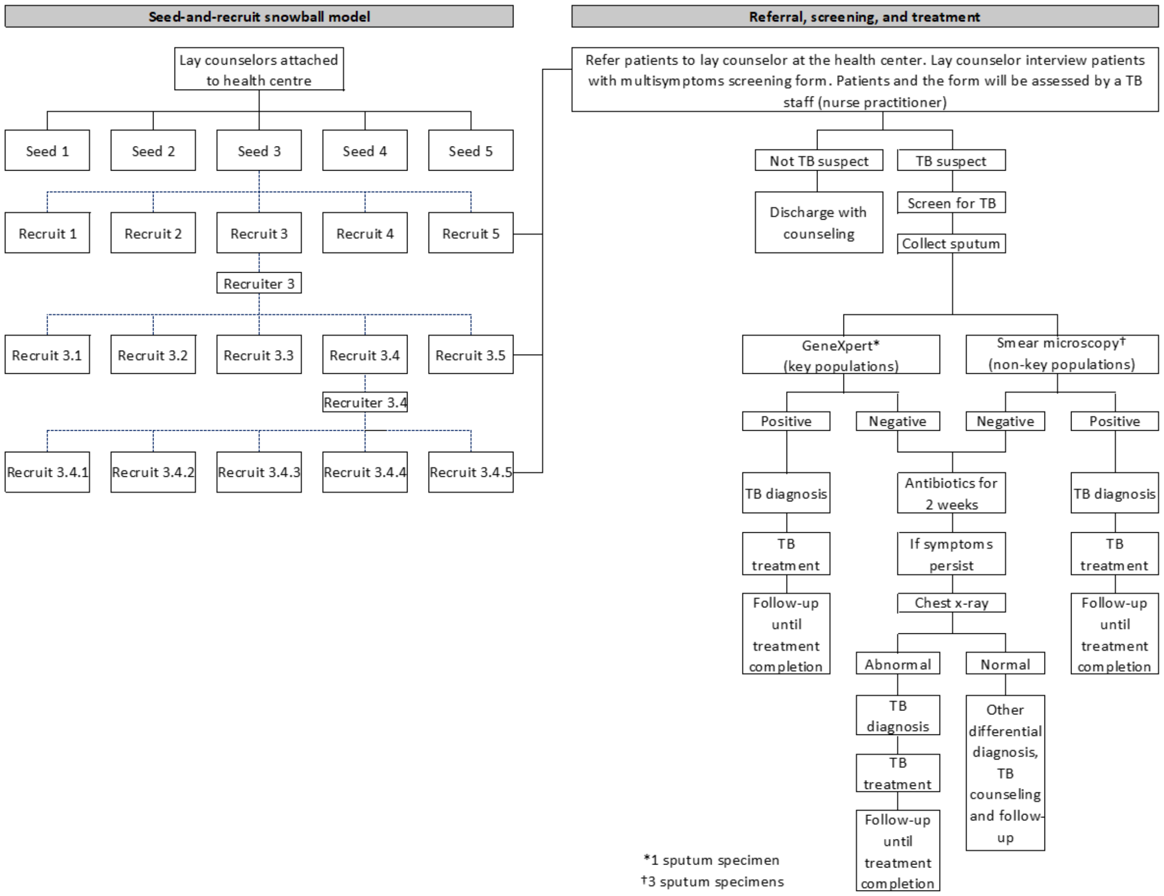

The operations team (field officers and lay counselors) from KHANA actively sought seeds—TB survivors and their TB-affected family members and other key informants in the community. The seeds were trained to find people with presumptive TB in the community, especially among key populations (KP) for TB (people aged 55 and above, people with diabetes, people living with HIV, household contacts of TB patients, and people who use and inject drugs) [2], using a symptom assessment questionnaire and refer those who might have TB to the health centers for TB workup. New recruiters were identified among those who were newly diagnosed with TB (eligibility criteria: undergoing treatment for TB and deemed non-infectious by a clinician, has knowledge of TB symptoms, and willing to volunteer) to find other people with presumptive TB in the community. These processes were carried out in a snowball approach, as illustrated in the left panel of **Supplementary Figure S1**. People with presumptive TB that were referred to the health centers were further examined by a nurse practitioner. Sputum samples from people with presumptive TB were collected and evaluated at the health centers using smear microscopy (non-KP) or GeneXpert® MTB/RIF system (KP). TB was diagnosed by the clinicians and managed following the national TB guideline.[3]

Supplementary Figure S2: One-off roving ACF.

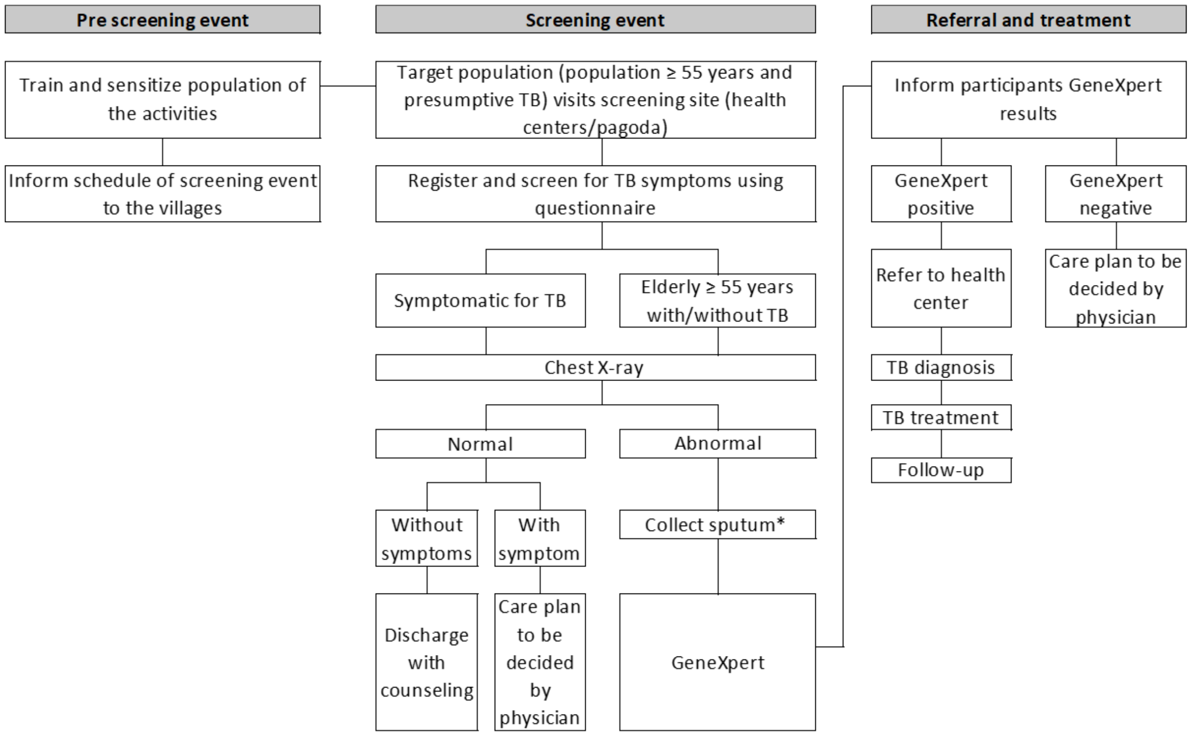

*1 sputum specimen

The one-off roving ACF model was conducted by CATA at a designated time, day, and place in the community (health centers or pagodas), targeting persons aged ⩾ 55. To ensure sufficient coverage and reach, the screening events were planned according to the number of health centers and the proximity between villages in each OD. The event was made known to the communities through the village health support groups, and individuals who visited the event were screened using a tablet-based TB symptom questionnaire. All persons exhibiting TB symptoms and all individuals aged ⩾ 55 regardless of symptoms were subjected to chest X-ray (CXR) examinations. Individuals with abnormal CXR suggestive of TB were further assessed using GeneXpert® MTB/RIF system available on-site. People who were newly diagnosed with TB at the screening sites were referred for treatment and follow-up at the health centers.

Cost per test or sample incurred at the health centers (diagnostic and medical procedures)

Supplementary Table S1: Breakdown of the health system cost: diagnostic and medical procedures.

Diagnostics and medical procedures*	Min (USD†)	Max (USD†)	Average (unit cost) (USD†)	Frequencies (ACF using seed-and-recruit model)‡	Total cost (USD†)
GeneXpert MTB/RIF	37.3	40.9	39.0	5265	205488
Consultation and clinical examination	1.3	2.0	1.7	12074	20312
Smear microscopy§					
Fluorescent	1.7	2.1	1.9	5339	8821
Ziehl–Neelsen	NA	1.4	1.4		
Liquid culture	12.8	25.1	19.0	2	38
Other follow-up work on positive culture results and identification of Mycobacterium tuberculosis complex	12.6	16.2	14.4	2	29
Drug susceptibility testing for individuals suspected of drug-resistant TB	NA	48.7	48.7	5	243
Total (health system cost for ACF using seed-and-recruit model)‡					234931

TB: tuberculosis, ACF: active case finding, USD: United States Dollar: MTB/RIF: Mycobacterium tuberculosis/resistance to rifampicin, NA: data not available

*Costs for chest radiographs and sputum samples transportation have been included in the program cost (refer to Table 3).

†Adjusted for inflation and presented in 2018 USD.

‡As health system costs are negligible for one-off roving ACF, only data from ACF using a seed-and-recruit model are presented in this table.

§The higher cost between fluorescence and Ziehl–Neelsen smear microscopy was selected (USD 1.9 per test) as we lacked data on the number of respective examinations performed.

Estimation of the number of undetected cases in the control sites

Passive case finding (PCF) is the default setup in the national health system in Cambodia. Therefore, we assumed that the TB cases notified by each operational district (intervention sites) to CENAT that were not accounted for by ACF were otherwise detected via PCF. Subsequently, we computed the median proportion of cases detected via PCF in the intervention sites (0.384, **Supplementary Table S2**) and applied it to the control sites to estimate the number of undetected TB cases that could have been identified by ACF should the interventions were implemented accordingly. The total number of undetected cases in the control sites was estimated to be 4615 (**Supplementary Table S3**).

Supplementary Table S2: Estimated proportion of TB cases detected via PCF in the intervention sites.

Operational districts	Proportion of cases detected via PCF
Sampov Luon	0.367
Steung Treng	0.421
Angkor Chey	0.344
Kampong Trach	0.466
Samraong	0.305
Kamchay Mear	0.100
Sitho Kandal	0.438
Koh Thom	0.435
Romeas Hek	0.445
Ponhea Krek	0.191
OuDaung	0.401
Koh Andet	0.318

TB: tuberculosis, PCF: passive case finding

Supplementary Table S3: Estimated number of undetected cases in the control sites that could have been identified by ACF if implemented.

Operational districts	Estimated number of undetected TB cases
Stoung	289
Steung Trong	323
Saang	451
Kampot	449
Kratie	429
Peam Ro	302
Srei Santhor	292
Ksach Kandal	377
Boribo	632
Memut	422
Phnom Srouch	376
Krakor	273

TB: tuberculosis, PCF: passive case finding

Costs of PCF and C-DOTS

Cost of PCF used in the main analysis

We estimated the cost incurred at the health facilities to screen and diagnose 12074 people with presumptive TB identified by KHANA to be USD 247886 (sum of USD 234931—health system cost of ACF using seed-and-recruit model and USD 12955—costs of chest radiographs and sputum transportation included in the program cost). Therefore, the estimated cost per person tested at the health facilities was USD 20.53 (USD 247886/12074). To estimate the cost of PCF, we assumed that a similar proportion of people who self-initiated care-seeking at the health facilities (PCF) would undergo similar processes as per the people with presumptive TB identified and referred by ACF using a seed-and-recruit model to the health facilities for TB work-up. Therefore, the diagnostic and medical procedure cost of PCF was obtained by multiplying USD 20.5 by the number of cases notified by PCF in the intervention and control sites. Treatment/ community directly observed treatment short-course (C-DOTS) cost was also accounted for in the computation of PCF cost. We multiplied the estimated C-DOTS cost per person (USD 65.35) [4] with the total number of TB cases notified by PCF (assumption: all people with TB identified via PCF initiated and completed TB treatment). Hence, the total PCF costs in the intervention and control sites were USD 188158 and USD 246898, respectively.

Costs of PCF estimated from published studies

We investigated two scenarios for the cost associated with PCF. First, we referred to the 715 individuals who attended the health center in the PCF arm in the study reported by Yadav and colleagues[5] as individuals screened for TB. Of the 715 individuals, 396 were labeled as treated, which we presumed as diagnosed with TB and started on treatment. Therefore, the estimated TB detection rate was 54.6%, and we utilized this estimate to approximate the total number of individuals screened for TB in the control sites (total TB cases detected and notified: 3470. Estimated number of people screened: 6355). The authors reported that the total cost of PCF was USD 54000. Considering the cost was for screening, diagnosis of TB, and DOTS, we worked out the cost per person to be USD 83.02 (adjusted for inflation). In this study, we only considered the actual number of cases notified in the control sites in estimating the total cost of PCF because 1) we do not have the actual data as to how many individuals were screened by PCF in the control sites, and 2) the number of people screened would be larger (8487) than the eventual diagnoses that were notified. We took a conservative approach as over-inflation of PCF cost would naturally reduce the net costs (the nominator in ICER calculations) and potential bias in favor of the interventions. Therefore, the estimated costs of PCF in this scenario in the intervention and control sites are USD 181890 and USD 238674 (adjusted for inflation), respectively.

Second, we considered another scenario with the cost presented by Pichenda and colleagues [6] in 2012. The study evaluated the cost of TB from the onset of the disease until treatment completion between 2008 and 2009 at the health facilities that have adopted the national TB control strategy and provided DOTS. We extracted only the cost of consultation and medicine before TB diagnosis (USD 65.4 per person; adjusted for inflation) and regarded it as the cost of TB care-seeking at the health facilities. As direct medical costs during treatment were reported as 0 and not included in our analysis, we undertook the approach of estimating the treatment cost under the sub-section "cost of PCF used in the main analysis" described above. Therefore, the estimated cost of PCF in this scenario was USD 286469 and USD 375900 in the intervention and control sites, respectively.

Using these estimates, the cost per DALY averted for ACF using a seed-and-recruit model, one-off roving ACF, and TB REACH are illustrated in **Supplementary Table S4**.

Supplementary Table S4: Sensitivity analyses of the different costs of PCF and the resultant incremental cost-effectiveness ratio.

Interventions	Variations of PCF costs (USD)	Cost in USD per DALY averted (95% uncertainty interval) 1*	2†
	Intervention sites	181890	286469
	Control sites	238674	375900
ACF using a seed-and-recruit model		257 (251–265)	246 (238–259)
One-off roving ACF		205 (197–213)	192 (183–206)

TB: tuberculosis, DALY: disability-adjusted life years, PCF: passive case finding, ACF: active case finding, USD: United States Dollar

* Cost of PCF extracted from a study by Yadav and colleagues conducted in Cambodia in 2014 (adjusted for inflation and presented in USD 2018).

†Cost of PCF extracted from a study by Pichenda and colleagues conducted in Cambodia in 2012 and Pallas and colleagues (2014) (adjusted for inflation and presented in USD 2018).

<u>Cost of C-DOTS</u>

A previously published study by Yadav and colleagues also reported the cost of TB treatment (C-DOTS) in their model. The study reported a range between USD 220 and USD 330, with the best estimate of USD 275 (adjusted for inflation). We included this information in our one-way sensitivity analysis, and the resultant ICER for ACF using a seed-and-recruit model (range: USD 302–334) and one-off roving ACF (USD 297–330) remained below the cost-effective thresholds.

Sensitivity analysis of the variation of disability-adjusted life years of the ACF models if the models were not implemented concurrently and the resultant incremental cost-effectiveness ratio

As both ACF using a seed-and-recruit model and one-off roving ACF were implemented synchronously in the intervention sites, we further explored the potential impact on TB case detection if only one intervention was implemented. We did not observe a definitive trend that concurrent implementation has led to a change in the TB case detection of either model (Supplementary Figure S3). For ACF using a seed-and-recruit model, we examined the change pre-and post-implementation of one-off roving ACF in four sites (Kamchay Mear, Sitho Kandal, Koh Thom, and Romeas Hek) where data from two quarters pre- and post-implementation of one-off roving ACF was available. The changes in case detection range from 7% to 49%. For one-off roving ACF, we examined the differences between the maximum and the minimum number of TB cases detected in urban and rural ODs. In the urban ODs, the maximum number of TB cases detected was 271, and the minimum was 190 (difference: 30%). In the rural ODs, the maximum number of TB cases detected was 239, and the minimum was 132 (difference: 45%).

To explore the change in DALY, if the intervention was implemented in silo, we inflated the DALYs (wholly) for each intervention by 10% to 50%. We also inflated the health system cost by the same proportion. We assumed TB deaths to increase proportionally as well. The resultant ICER for ACF using a seed-and-recruit model (range: USD 272–349) and one-off roving ACF (USD 216–285) remained below the cost-effective thresholds.

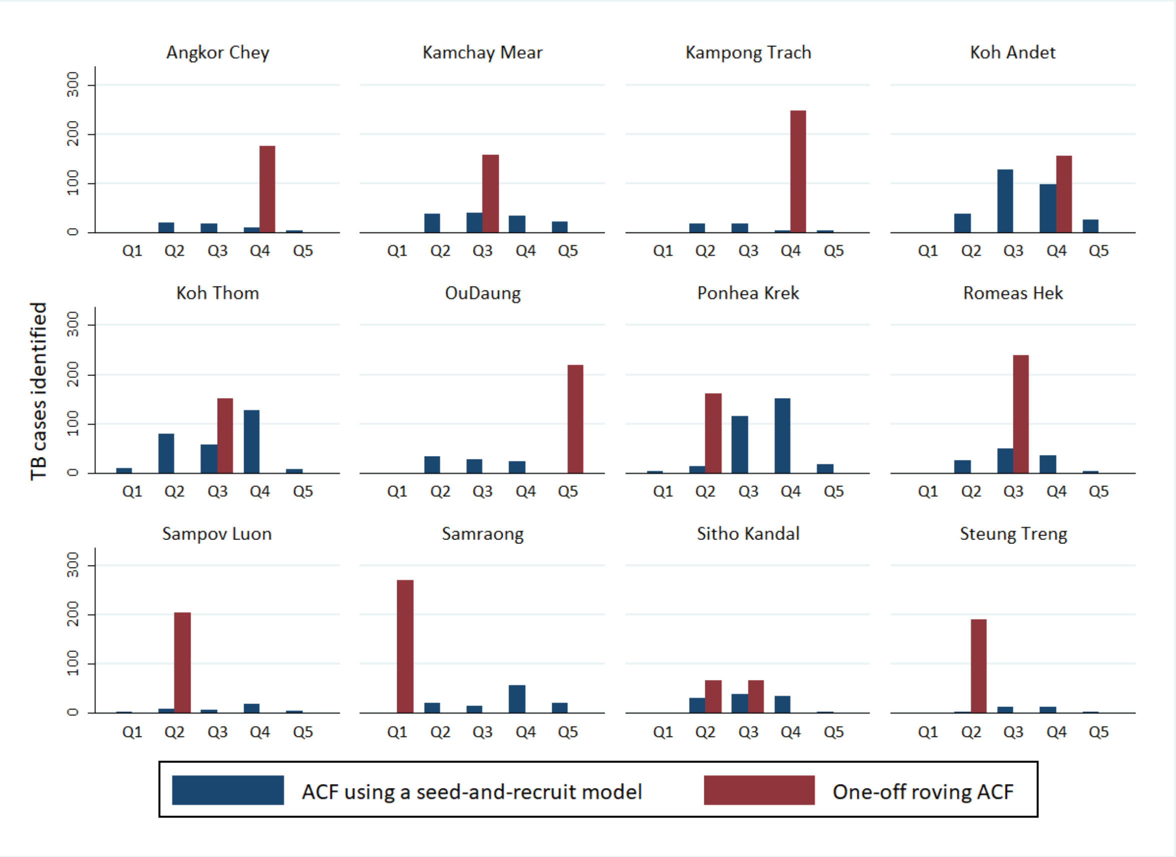

Supplementary Figure S3: Distribution of TB cases identified during the implementation period.
The x-axis shows the period of implementation from quarter 4 of 2018 to quarter 4 of 2019 (inclusive), which included a total of 5 quarters (indicated as Q1 to Q5 in the figure). The y-axis shows the total TB cases identified by the active case-finding models.

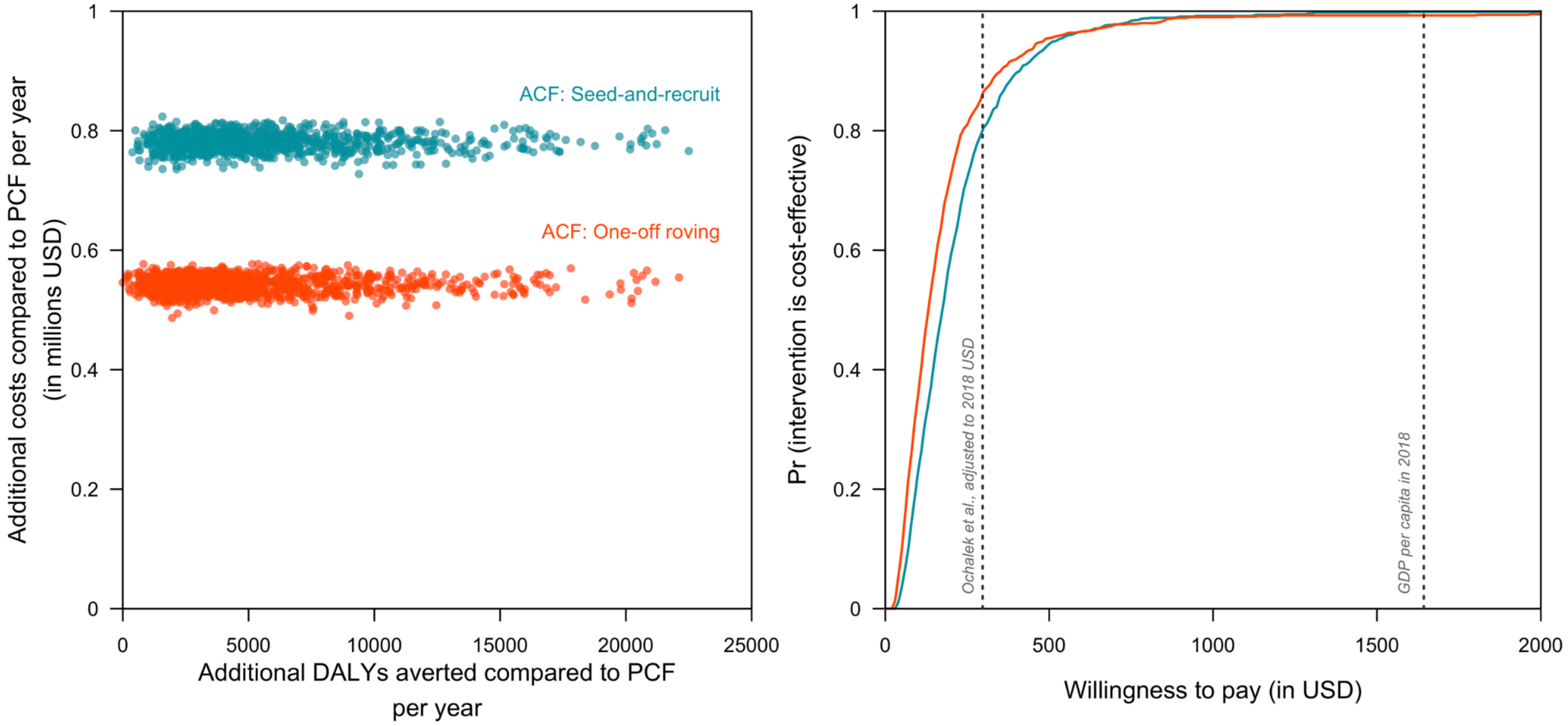

Supplementary Figure S4: Probabilistic sensitivity analysis: Incremental cost-effectiveness scatter plots and acceptability curves of two active-case finding interventions, seed-and-recruit and one-off roving, against the default passive case-finding model. The incremental cost-effectiveness scatterplot of the sensitivity analyses and acceptability curves for the seed-and-recruit active case-finding model versus passive case-finding model are presented as blue points and curves, respectively. The incremental cost-effectiveness scatterplot of sensitivity analyses and acceptability curves for the one-off roving active case-finding model versus passive case-finding model are presented as dark orange points and curves, respectively. Two cost-effectiveness thresholds—2018 GDP per capita and the conservative Ochalek's estimates—are included (broken lines) as references on the cost-effectiveness acceptability curves.

References

1. Teo AKJ, Prem K, Evdokimov K, Ork C, Eng S, Tuot S, et al. Effect of community active case-finding strategies for detection of tuberculosis in Cambodia: study protocol for a pragmatic cluster randomized controlled trial. Trials [Internet]. 2020 [cited 2020 Feb 25];21. Available from: https://trialsjournal.biomedcentral.com/articles/10.1186/s13063-020-4138-1

2. Yi S, Teo AKJ, Sok S, Tuot S, Tieng S, Khun KE, et al. Barriers in access to services and information gaps by genders and key populations in the national Tuberculosis programme in Cambodia. Global Public Health [Internet]. 2021 [cited 2021 Sep 25];1–14. Available from: https://www.tandfonline.com/doi/full/10.1080/17441692.2021.1954226

3. National Center for Tuberculosis and Leprosy Control (CENAT). Technical guidelines on tuberculosis control (2nd edition). Phnom Penh: Ministry of Health; 2016.

4. Pallas SW, Courey M, Hy C, Killam WP, Warren D, Moore B. Cost Analysis of Tuberculosis Diagnosis in Cambodia with and without Xpert® MTB/RIF for People Living with HIV/AIDS and People with Presumptive Multidrug-resistant Tuberculosis. Appl Health Econ Health Policy. 2018;16:537–48.

5. Yadav RP, Nishikiori N, Satha P, Eang MT, Lubell Y. Cost-effectiveness of a tuberculosis active case finding program targeting household and neighborhood contacts in Cambodia. Am J Trop Med Hyg. 2014;90:866–72.

6. Pichenda K, Nakamura K, Morita A, Kizuki M, Seino K, Takano T. Non-hospital DOT and early diagnosis of tuberculosis reduce costs while achieving treatment success. Int J Tuberc Lung Dis [Internet]. 2012 [cited 2020 Aug 11];16:828–34. Available from: http://www.ingentaconnect.com/content/10.5588/ijtld.11.0688
